# Interphase adhesion geometry is transmitted to an internal regulator for spindle orientation via caveolin-1

**DOI:** 10.1038/ncomms11858

**Published:** 2016-06-13

**Authors:** Shigeru Matsumura, Tomoko Kojidani, Yuji Kamioka, Seiichi Uchida, Tokuko Haraguchi, Akatsuki Kimura, Fumiko Toyoshima

**Affiliations:** 1 Department of Cell Biology, Institute for Virus Research, Kyoto University, Sakyo-ku, Kyoto 606-8507, Japan; 2 Advanced ICT Research Institute Kobe, National Institute of Information and Communication Technology, Nishi-ku, Kobe 651-2492, Japan; 3 Department of Chemical and Biological Science, Japan Women's University, Bunkyo-ku, Tokyo 112-8681, Japan; 4 Department of Pathology and Biology of Diseases, Kyoto University, Kyoto 606-8315, Japan; 5 Innovative Techno-Hub for Integrated Medical Bio-Imaging, Kyoto University, Kyoto 606-8501, Japan; 6 Faculty of Information Science and Electrical Engineering, Kyushu University, Nishi-ku, Fukuoka 819-0395, Japan; 7 Cell Architecture Laboratory, Structural Biology Center, National Institute of Genetics, Yata 1111, Mishima, Shizuoka 411-8540, Japan

## Abstract

Despite theoretical and physical studies implying that cell-extracellular matrix adhesion geometry governs the orientation of the cell division axis, the molecular mechanisms that translate interphase adhesion geometry to the mitotic spindle orientation remain elusive. Here, we show that the cellular edge retraction during mitotic cell rounding correlates with the spindle axis. At the onset of mitotic cell rounding, caveolin-1 is targeted to the retracting cortical region at the proximal end of retraction fibres, where ganglioside GM1-enriched membrane domains with clusters of caveola-like structures are formed in an integrin and RhoA-dependent manner. Furthermore, Gαi1–LGN–NuMA, a well-known regulatory complex of spindle orientation, is targeted to the caveolin-1-enriched cortical region to guide the spindle axis towards the cellular edge retraction. We propose that retraction-induced cortical heterogeneity of caveolin-1 during mitotic cell rounding sets the spindle orientation in the context of adhesion geometry.

## Abstract (web summary)

Studies imply that cell adhesion geometry during interphase dictates the orientation of the cell division axis. Here the authors show that accumulation of caveolin-1 to rapidly retracting regions during cell rounding sets the spindle orientation by recruiting Gαi1-LGN-NuMA to the cortex.

## Graphical abstract

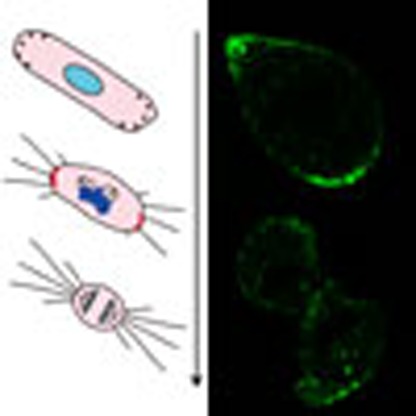

Spindle orientation plays an essential role in asymmetric cell division, tissue organization and organogenesis^1^,^2^,^3^. In asymmetrically dividing *Drosophila* neuroblasts, Bazooka, Par6 and atypical protein kinase C function as polarity proteins, while Inscuteable, Partner of Inscuteable (Pins), the trimeric G protein subunit Gαi, mushroom body defective (Mud) and Discs large regulate spindle orientation^4^,^5^. Pins interacts with cortically anchored Gαi and links astral microtubules with the cell cortex through motor proteins dynein and kinesin Khc-73 (refs 4, 5, 6, 7). This spindle orientation machinery is evolutionarily conserved in mammals^1^,^2^. In cultured mammalian cells, a dynein-binding protein NuMA^8^ (Mud in *Drosophila*) associates with LGN (Pins in *Drosophila*) during M phase. This association induces a conformational switch of LGN to an open state, allowing it to associate with Gαi at the cortex^9^. Then, the NuMA–LGN–Gαi complex at the cortex guides the spindle towards the polarity axes of cells.

Cells determine their division axis in response to not only intrinsic polarity proteins but also external cues, including cell–cell and cell-extracellular matrix (ECM) adhesions, through adhesion molecules such as cadherins and integrins^10^,^11^,^12^,^13^,^14^. We have previously shown that, in non-polarized adherent mammalian cells such as HeLa cells, spindles align along the cell–ECM adhesion plane to ensure attachment of both daughter cells to the substrate after cell division^11^. This spindle orientation depends on β1-integrin-mediated cell–substrate adhesion and requires regulators of the actin cytoskeleton and astral microtubules as well as signalling molecules including Abelson murine leukaemia viral oncogene homologue 1 (ABL1)^11^,^15^,^16^.

In addition to the planar spindle orientation relative to the surface of ECM, previous studies using an elegant micro-contact printing technique have shown that HeLa cells dividing on fibronectin-coated micropatterns orient their spindles relative to the pattern of cell–ECM adhesion geometry during interphase^17^,^18^,^19^. Adhesion geometry has been shown to impose a force field on the cortex of metaphase cells through actin filament-based retraction fibres, which eventually dictates the spindle orientation^20^. This spindle orientation requires the activity of Src kinase as well as Gαi1–LGN–NuMA-mediated dynein-dependent pulling forces on the cortex, which are regulated by signals from centrosomes or chromosomes^17^,^21^. However, it remains unclear when and how the geometrical cues are translated to cortical heterogeneity that governs spindle orientation. Here, we show that RhoA-dependent mitotic cell rounding generates caveolin-1-associated cholesterol-enriched membrane microdomains (CEMMs) at the retracting cellular edges and subsequently at the proximal end of retraction fibres. These domains are enriched with active RhoA, active β1-integrin and caveolin-1, and recruit the Gαi1/LGN/NuMA complex to direct the spindle axis towards the cortex enriched with retraction fibres. Our findings provide the molecular basis that links interphase adhesion geometry with spindle orientation regulators.

## Results

### Cell division axis correlates with cellular edge retraction

**Figure 1:**
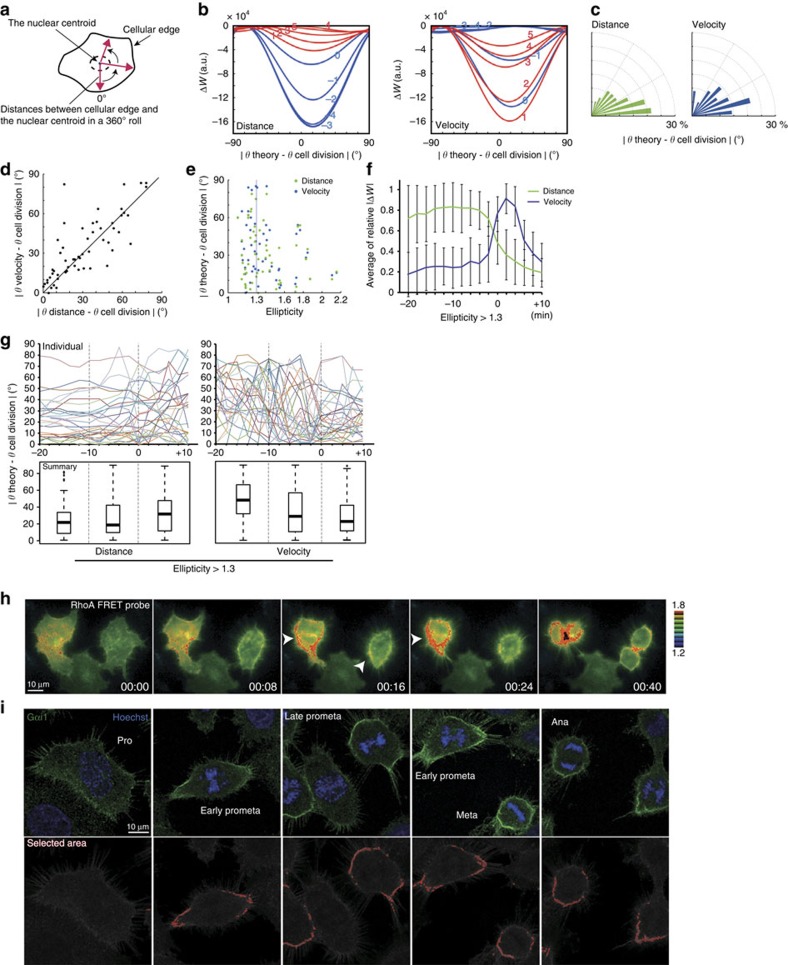
Velocity of cellular edge retraction during mitotic cell rounding is a cue that dictates the spindle orientation. (**a**) Scheme for measuring cellular geometry. (**b**) The theoretical potential energy landscape (Δ*W*_distance_ and Δ*W*_velocity_) of a representative example is shown. Blue and red lines represent Δ*W* before and after onset of mitotic cell rounding, respectively. The angle with the lowest Δ*W* is defined as the theoretical angle in each sample. Each number represents time points. (**c**) Angular distribution (*n*=55) of accuracy of the predicted spindle orientation. (**d**) Scatter plot comparing the accuracy of the predicted spindle orientation by distance- versus velocity-based methods in each sample. (**e**) Dot plot representing the prediction accuracy with respective ellipticity (ratio of the length of the longest/shortest cell axis). (**f**) Fold change of the average of ΔW at every 10 min (five frames) in cells with more (left) or less (right) than 1.3 ellipticity. Time 0 represents the onset of cell rounding (see Methods). (**g**) Angular difference between the theoretical and actual spindle angles at each time point in every sample (upper). Theoretical angles were calculated by distance- (left) or velocity-based (right) methods in cells with more than 1.3 ellipticity. Box plots representing the angles for every 10 min (five frames) are shown at the bottom. (**h**) Time-lapse serial ratio images of the RhoA FRET probe expressed in HeLa cells. (**i**) Images of Gαi1 (green) and Hoechst (blue) in HeLa cells from prophase to anaphase. A region with higher Gαi1 intensity than a certain threshold is represented as an enclosed red area at the bottom.

To determine cellular dynamicity that correlates with the spindle orientation, we analysed the temporal transition of adhesive geometry from G2 phase to anaphase. Time-lapse images of green fluorescent protein (GFP)-expressing HeLa cells cultured on a fibronectin-coated dish were obtained every 2 min, and the distances, Dis(*θ*), were measured between the nuclear centroid and cellular edge at each angle (1° interval) (Fig. 1a) for every time point. From the images, we predicted the most probable angle of spindle orientation based on a method developed in previous studies^18^,^19^. In this ‘distance-based prediction', the forces acting on the spindle poles are proportional to the distance from the cortex. We calculated the net torque exerted on the spindle and estimated the relative energy landscape (Δ*W*_distance_, normalized to the highest energy), where the most probable angle of spindle orientation corresponds to the angle that gives the minima of Δ*W*_distance_ (Fig. 1b, distance). The accuracy of the prediction was higher than that of random prediction (*P*=0.001, by binomial test): the predicted angles fell within 30° of the vicinity of the cell division axis (0 on the *X* axis in Fig. 1b) in 55.4% of cells (Fig. 1c, distance). It has been demonstrated experimentally and theoretically that, if the pattern of adhesion to the ECM is isotropic, the spindle orientation is almost random in cells without a clear long axis during interphase (that is, low ellipticity or low |Δ*W*_distance_|)^18^,^19^. Consistently, the accuracy of our ‘distance-based prediction' was high for cells with an elongated shape (65.5% of cells with more than 1.3 ellipticity), while only in 44.4% of cells with less than 1.3 ellipticity (Fig. 1e, distance). Importantly, after the onset of mitotic cell rounding, the ellipticity and |Δ*W*_distance_| decreased (Fig. 1f and Supplementary Fig. 1b, distance) and the predicted spindle angles became unreliable even in cells with an elongated shape during interphase (Fig. 1g and Supplementary Fig. 1c, distance). This result suggests that the cell shape no longer dictates spindle orientation after cell rounding. Therefore, we hypothesized that adhesion geometry is somehow transmitted through cellular signal transduction during mitotic cell rounding.

To gain an insight into the cellular signal transduction, we focused on cellular edge retraction during mitotic cell rounding. We measured the velocity of cellular edge retraction for each angle at each time point (V(*θ*, *t*)), which is defined as V(*θ*, *t*)=(Dis(*θ, t*)—Dis(*θ*, *t*−3))/6 min. We performed the same calculation as in the distance-based prediction except that we assumed the forces acting on the spindle poles were proportional to V(*θ*, *t*), instead of Dis(*θ*, *t*), and calculated the torque and relative energy landscape (Δ*W*_velocity_). We named this calculation ‘velocity-based prediction' (Fig. 1b, velocity). The prediction accuracy was higher than in the random prediction (*P*=0.011, by binomial test), and the same as in distance-based predictions (Fig. 1c–e, velocity). It is noteworthy that the prediction accuracies using velocity defined by 2 or 10 min intervals were similar (Supplementary Fig. 1a), indicating the robustness of this calculation. Importantly, in contrast to |Δ*W*_distance_|, |Δ*W*_velocity_| displayed a peak immediately after the onset of mitotic cell rounding (Fig. 1b,f, and Supplementary Fig. 1b, velocity). Consequently, the prediction by cellular edge retraction became more accurate and converges after the onset of mitotic cell rounding (Fig. 1g). These results suggest that interphase adhesion geometry is transmitted to the spindle orientation machine via a signalling pathway that is involved in cellular edge retraction during mitotic cell rounding. In support of this idea, when the spindle angle was calculated by ‘surface-based prediction', where forces acting on the spindle poles are assumed to be proportional to the cellular edge length between each angle (1° interval), the accuracy of prediction was again the same as in distance/velocity-based predictions (Supplementary Fig. 1b, surface). This result suggests that molecules or membrane components gathering in the retracting area during mitotic cell rounding may function in spindle orientation.

### RhoA activates in the retracting area during cell rounding

**Figure 2:**
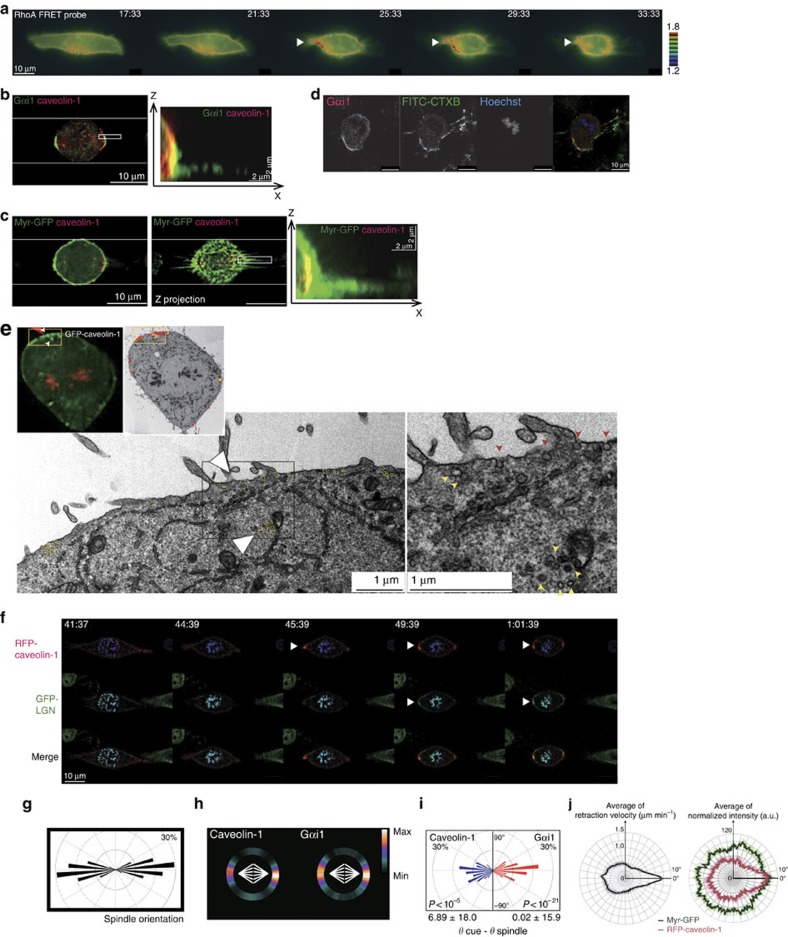
Caveolin-1 accumulates in the rapidly retracting cellular edge during mitotic cell rounding. (**a**) Time-lapse serial ratio images of the RhoA FRET probe expressed in HeLa cells cultured on a fibronectin-line micropattern. (**b**) Images of a metaphase cell cultured on the line micropattern stained with anti-Gαi1 (green) and anti-caveolin-1 (red) antibodies. A middle section of Z-stack images (left) and an X-Z projection image in the boxed area (right) are shown. (**c**) Images of a metaphase cell expressing MyrPalm-GFP (green) cultured on the line pattern stained with an anti-caveolin-1 antibody (red). A middle section of Z-stack images (left), a Z projection image (middle), and an X-Z projection image in the boxed area (right) are shown. (**d**) Images of a mitotic cell cultured on a fibronectin-coated coverslip and stained with an anti-Gαi1 antibody, FITC-labelled cholera toxin subunit B (CTX-FITC), and Hoechst. (**e**) Images of GFP-caveolin-1 fluorescence and electron microscopy. The boxed area is enlarged (middle and right). Red dots and arrowheads represent caveola-like structures, and yellow dots and arrowheads represent caveosome-like structures. (**f**) Time-lapse images of a mitotic cell expressing GFP-LGN (green), RFP-caveolin-1 (red) and CFP-histone H1 (blue) on the line micropattern. Arrowheads indicate the recruitment of fluorescent proteins at the retracting cortical region. (**g**) Angular distributions of the spindle orientation along the long axis of the line pattern. (**h**) The distribution of cortical caveolin-1 or Gαi1 signals against the spindle axis (white). The colour-code indicates the average of the relative intensity at each angle. (**i**) Distribution of the angle difference between the spindle angle and predicted spindle orientation based on the cortical intensity of caveolin-1 or Gαi1. *P* values represent the probability against random prediction (within 30°). Mean±s.d. (below). (**j**) Distribution of retraction velocity per angle (left) and distribution of values per angle at the cellular edge (right). For details, see Supplementary Figs 3 and 4.

To gain insight into the underlying mechanisms, we examined the activity of a small GTPase RhoA, which governs mitotic cell rounding^22^,^23^. The activity of RhoA, which can be detected by a fluorescence resonance energy transfer (FRET)-based sensor probe for RhoA activity^24^,^25^, increased at the onset of mitosis (Fig. 1h) as reported previously^25^. Interestingly, RhoA was preferentially highly activated at the rapidly retracting cellular edge during mitotic cell rounding, followed by alignment of the spindle axis towards the RhoA-activated field during prometa/metaphase (Fig. 1h, arrowheads). Concurrently with the local activation of RhoA, the spindle orientation regulator Gαi1 was recruited to the retracting cellular edge before determination of the spindle orientation (Fig. 1i). The correlation between the retraction-associated local activation of RhoA and Gαi1 recruitment to the retracting cellular edge was obvious when the cells were plated on a line-shaped fibronectin micropattern (Fig. 2a,b) on which mitotic spindles are known to be aligned along the long axis of the pattern^17^,^18^ (Fig. 2g). Active RhoA was present throughout the cortex during interphase (Fig. 2a, 17:33−21:33). However, at the onset of cell rounding, RhoA was highly activated at the rapidly retracting cellular edge (Fig. 2a, 25:33−33:33, arrowheads). Concurrently, the phosphorylation level of myosin II, which occurs downstream of the RhoA-ROCK signalling pathway^26^, was accumulated at the retracting cellular edge in a RhoA-dependent manner (Supplementary Fig. 2). Simultaneously, Gαi1 was present along the retraction fibres and on the mitotic cell cortex at the proximal end of the retraction fibres in metaphase cells (Fig. 2b). Active RhoA and Gαi1 were barely detected at the cortex region facing the non-adhesive area during prometa/metaphase (Fig. 2a,b). These results suggest that RhoA-dependent cell edge retraction generates the cortical heterogeneity that induces the accumulation of Gαi1 at the proximal end of the retraction fibres in the retracting cortical area during mitotic cell rounding.

### Caveolin-1-associated CEMMs assemble at the retracting area

Active RhoA and Gαi1 localize in CEMMs including lipid rafts and caveolae^27^,^28^,^29^. Thus, we next examined enrichment of CEMM components at the retracting cellular edge during mitotic cell rounding. In prometa/metaphase cells, we found that the CEMM component ganglioside GM1 (refs 30, 31), which can be detected by fluorescein-5-isothiocyanate (FITC)-labelled cholera toxin subunit B (CTXB)^32^,^33^, was co-localized with Gαi1 on the cell cortex (Fig. 2d). In addition, caveolin-1, an essential component of caveola microdomains^31^, was co-localized with Gαi1 at the proximal end of the retraction fibre in metaphase cells on the line pattern (Fig. 2b,c). During mitotic cell rounding, it has been reported that a large amount of caveolae is internalized from the plasma membrane to the intracellular compartments when the cells are seeded on uncoated coverslips^34^. However, when the cells were cultured on fibronectin, we found pronounced levels of caveolin-1 at the cortex in addition to the intercellular compartments in mitotic cells (Fig. 2b,c; Supplementary Movie 1). In addition, a correlative light and electron microscopy image^35^ of mitotic HeLa cells stably expressing GFP-tagged caveolin-1 (HeLa-GFP-caveolin-1) displayed clusters of caveola-like structures on the cortex as well as vesicles in the vicinity of the cell cortex in GFP-caveolin-1-positive areas (Fig. 2e). These results suggest that caveolin-1-associated CEMMs are assembled on the plasma membrane at the proximal end of the retraction fibres in mitotic cells on the fibronectin ECM.

To investigate the dynamics of caveolin-1 and spindle orientation machineries during mitotic cell rounding, we performed time-lapse imaging of HeLa cells expressing red fluorescent protein-tagged caveolin-1 (RFP-caveolin-1), GFP-tagged LGN (GFP-LGN), and cyan fluorescent protein-tagged histone H1 (CFP-H1) on the line pattern (Fig. 2f). The obtained images showed that RFP-caveolin-1 was recruited to the rapidly retracting cellular edge at the onset of mitotic cell rounding before GFP-LGN recruitment to the retracting cellular edge (Fig. 2f). Furthermore, Gαi1 and caveolin-1 were accumulated on the cortex proximal to the spindle poles (Fig. 2g,h). In addition, the cortical distribution of both Gαi1 and caveolin-1 was able to precisely predict the spindle orientation on the line pattern by the use of ‘cortical cue intensity-based prediction'^36^, whereby the angular distribution of spindle orientation is theoretically predicted by calculating the torque on the spindle exerted by astral microtubules with a force proportional to the intensity of the cortical cues (that is Gαi1 or caveolin-1; Fig. 2i). Moreover, the prediction accuracy within 30° was much higher compared to the random prediction (*P*<10^−5^ in caveolin-1 and *P*<10^−21^ in Gαi1, respectively, by binomial test). These results support the notion that cortical Gαi1 and/or caveolin-1 dictates spindle orientation. Notably, the time-lapse imaging of HeLa cells expressing RFP-caveolin-1 and plasma membrane marker myristoylated-GFP (Myr-GFP) (Supplementary Movie 2), together with quantification analyses of the retraction velocity and cortical signal accumulation of RFP-caveolin-1 and Myr-GFP as a function of angular coordination (Supplementary Fig. 3), showed that the retraction velocity closely correlated with the cortical accumulation of RFP-caveolin-1, but poorly with that of Myr-GFP (Fig. 2j; Supplementary Fig. 4a). The correlation was also observed between the retraction velocity and cortical accumulation of GFP-LGN (Supplementary Fig. 4c). Importantly, when the cells were cultured on a soft polyacrylamide gel (12%) coated with fibronectin, the correlation between the retraction velocity and cortical signal accumulation of RFP-caveolin-1 was decreased significantly compared with that in cells cultured on a fibronectin-coated normal coverslip, even though the averages of the maximum and minimum retraction velocities were almost the same as those in the controls (Supplementary Fig. 4a,b; Supplementary Movie 3). Thus, retraction speed itself is not a cue for spindle orientation, rather gathering the membrane components, including cell–ECM adhesion-related molecules, to the retracting cell edge during mitotic cell rounding likely functions as a cue for spindle orientation. Taken together, these results raise the possibility that the anisotropic activation of RhoA at the retracting cellular edge during mitotic cell rounding transmits the adhesion geometry to Gαi1/LGN by accumulating caveolin-1-enriched CEMMs at the cortex enriched with retraction fibres to determine the spindle orientation in the context of the ECM geometry.

### RhoA pathway is required for caveolin-1 accumulation

**Figure 3:**
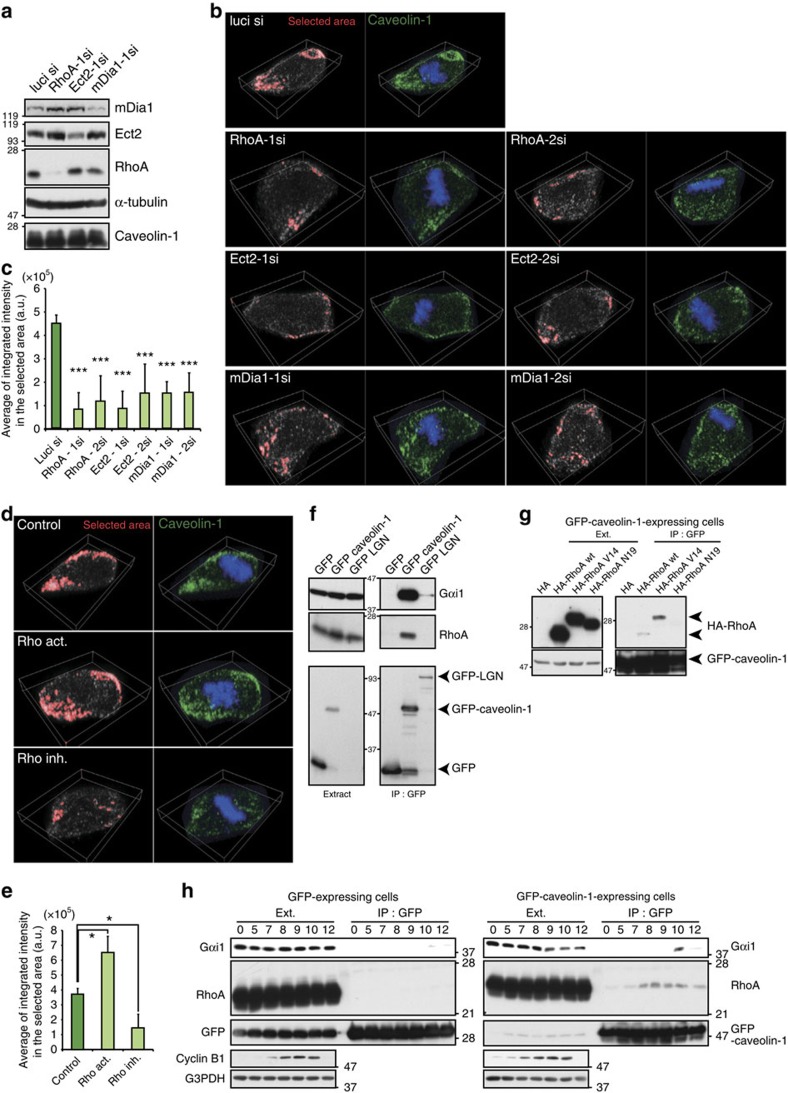
Cortical recruitment of caveolin-1 depends on the RhoA signalling pathway. (**a**) Western blot analysis of mDia1, Ect2, RhoA, caveolin-1 and α-tubulin (control) in the cells in **b**. (**b**) Three-dimensional reconstituted images of prometaphase cells and metaphase cells with cell rounding defects, which were treated with two independent RhoA siRNAs, Ect2 siRNAs, mDia1 siRNAs, or control luci siRNA, and stained with an anti-caveolin-1 antibody (green) and Hoechst (blue). The region with a caveolin-1 intensity higher than a certain threshold is represented as the enclosed red area in the left panel. (**c**) Average of the integrated caveolin-1 intensity in the red region in **b** was measured using z-stack images for each sample (mean±s.d. from three independent experiments; *n*>20/experiment) (**d**) Three-dimensional reconstituted images of prometaphase cells treated with or without a Rho inhibitor or activator and stained with an anti-caveolin-1 antibody (green) and Hoechst (blue). The region with the caveolin-1 intensity higher than a certain threshold is represented as the enclosed red area in the left panel. (**e**) Average of the integrated caveolin-1 intensity in the red region in **d** was measured using z-stack images for each sample (mean±s.d. from three independent experiments; *n*>20/experiment). (**f**) Total lysates from mitotically arrested cells expressing GFP, GFP-caveolin-1, or GFP-LGN were subjected to immunoprecipitation with an anti-GFP antibody and subjected to western blotting with anti-GFP, anti-RhoA and anti-Gαi1 antibodies. (**g**) Co-immunoprecipitation analysis of exogenously expressed HA-RhoA wt, HA-RhoA V14 (constitutively active form), or RhoA N19 (dominant negative form) with GFP-caveolin-1 in mitotically arrested cells. (**h**) Co-immunoprecipitation analysis of endogenous RhoA or Gαi1 with GFP-caveolin-1 or control GFP in synchronized cells released from the double thymidine block. *P<0.05 and ***P<0.001, analysed by Dunnett's multiple-comparison test.

To investigate the aforementioned possibility, we first tested the requirement of RhoA for caveolin-1 recruitment to the cell cortex during mitotic cell rounding. Depletion of RhoA with two independent small interfering RNAs (siRNAs) suppressed mitotic cell rounding as reported previously^22^,^23^. Furthermore, RhoA-depletion suppressed the accumulation of caveolin-1 on the cell cortex without affecting the expression level of caveolin-1 protein (Fig. 3a–c; Supplementary Fig. 5). Similar results were obtained when the cells were depleted of the RhoA guanidine-exchanging factor Ect2 (ref. 23; (Fig. 3a–c). In addition, treatment of the cells with RhoA inhibitory peptides for 1 h at the onset of mitosis was sufficient to suppress mitotic cell rounding and cortical accumulation of caveolin-1 (Fig. 3d,e, Rho inh.). This result indicated that activation of RhoA at the onset of mitosis, but not during interphase, is required for both mitotic cell rounding and cortical accumulation of caveolin-1. Notably, similar to knockdown of RhoA, depletion of the RhoA effector mDia1 (refs 37, 38) in cells suppressed mitotic cell rounding and cortical accumulation of caveolin-1 (Fig. 3a–c), suggesting positive feedback signals between RhoA and mDia1 as reported previously^39^. Furthermore, RhoA-activating peptide treatments enhanced the accumulation of caveolin-1 on the cortex at the retracting cellular edge (Fig. 3d,e, Rho act.). These results demonstrate that caveolin-1 is recruited to the retracting cellular edge in a RhoA activity-dependent manner.

We next examined the association between caveolin-1 and RhoA in M phase-arrested cells. Using HeLa cells stably expressing GFP-caveolin-1, we found that GFP-caveolin-1 was co-immunoprecipitated with endogenous RhoA in M phase-arrested cells (Fig. 3f). In addition, GFP-caveolin-1 was found to associate preferentially with the active form of RhoA (RhoA V14) rather than wild-type RhoA, and barely co-immunoprecipitated with the dominant negative form of RhoA (RhoA N19) (Fig. 3g). Furthermore, the co-immunoprecipitation efficiency of GFP-caveolin-1 with endogenous RhoA was increased during M phase in synchronized cells (Fig. 3h). Importantly, GFP-caveolin-1 was co-immunoprecipitated with not only RhoA but also Gαi1 in M phase-synchronized cells (Fig. 3f,h). These results support the notion that caveolin-1 is closely associated with active RhoA and Gαi1 within the CEMM at the retracting cellular edge during mitotic cell rounding.

### β1-integrin pathway is required for caveolin-1 accumulation

**Figure 4:**
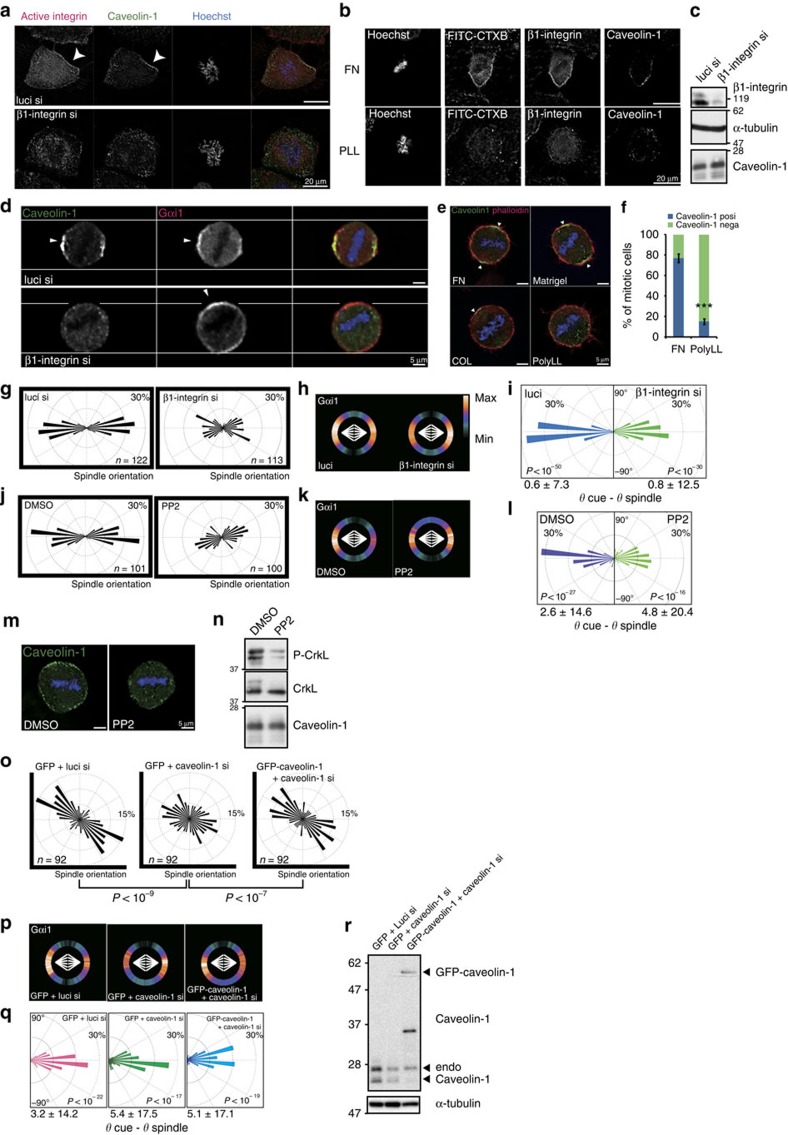
Caveolin-1 provides the link between ECM-derived external cues and Gαi1–LGN–NuMA-dependent spindle orientation machineries. (**a**) Images of prometaphase cells treated with luci siRNA or β1-integrin siRNA and then stained with anti-active β1-integrin (red) and anti-caveolin-1 (green) antibodies and Hoechst (blue). (**b**) Images of mitotic cells cultured on fibronectin-coated coverslips (upper panels) or poly-L-lysine-coated coverslips (bottom) and then stained with anti-active β1-integrin and anti-caveolin-1 antibodies, FITC-CTXB and Hoechst. (**c**) Western blot analysis of β1-integrin, caveolin-1 and α-tubulin (control) in cells treated with luci siRNA or β1-integrin siRNA. (**d**) Images of metaphase cells on the line pattern treated as in **c** and stained with anti-Gαi1 (red) and anti-caveolin-1 (green) antibodies, and Hoechst (blue). Arrowheads indicate the centre of caveolin-1 or Gαi1 crescents on the cortex. (**e**) Images of metaphase cells cultured on fibronectin, Matrigel, collagen (COL), or poly-L-lysine (PolyLL) and then stained with an anti-caveolin-1 antibody (green), RITC-labelled phalloidin (red), and Hoechst (blue). Arrowheads indicate the centre of the caveolin-1 crescent on the cortex. (**f**) Percentage of mitotic cells with or without caveolin-1 recruitment at the cell cortex. ***P<0.001, analysed by a *t*-test. (**g**,**j**,**o**) Angular distributions of the spindle orientation on the line pattern (**g**,**j**) or L-pattern (**o**). (**o**) Kolmogorov–Smirnov test (*P* values). (**h**,**k**,**p**) The distribution of cortical Gαi1 signals against the spindle axis (white). The colour-code indicates the average of the relative intensity at each angle. (**i**,**l**,**q**) Distribution of prediction accuracy as a subtracted angle between torque calculated angle and spindle angle. *P* values represent the probability against random prediction (within 30°). Mean±s.d. (below). (**m**) Images of a metaphase cell treated with PP2 (10 μM) or the control (DMSO) and then stained with an anti-caveolin-1 antibody (green) and Hoechst (blue). (**n**) Western blot analysis of caveolin-1, CrkL, and phospho-CrkL in cells in **m**. (**r**) Western blot analysis of caveolin-1 and α-tubulin (control) in cells in **o**, **p** and **q**.

Retraction fibres maintain connections between cells and the ECM during mitosis, and β1-integrin plays a major role in the cell–ECM adhesion of HeLa cells^11^. Thus, we next examined whether β1-integrin signalling is required for the localization of caveolin-1 to the cell cortex during mitotic cell rounding. In prometa/metaphase cells, caveolin-1 was co-localized with the active form of β1-integrin on the cortex where FITC-CTXB signals were enriched, indicating the accumulation of these proteins within the CEMM (Fig. 4a,b; Supplementary Movie 4). Depletion of β1-integrin by RNA interference (RNAi) had only a minor effect on the expression level of caveolin-1 protein, but suppressed its cortical localization in prometa/metaphase cells (Fig. 4a,c,d). In addition, plating the cells on poly-L-lysine, which inhibits integrin-dependent cell–ECM adhesion^40^ but did not inhibit RhoA-dependent cell rounding as was indicated by the comparable phosphorylation level of myosin II (Supplementary Fig. 2), reduced the cortical localization of caveolin-1. In contrast, plating the cells on fibronectin, Matrigel and collagen, resulted in proper cortical localization of caveolin-1 in prometa/metaphase cells (Fig. 4b,e,f). Furthermore, when the cells were treated with the Src family inhibitor PP2 (ref. 41), which abrogates ECM-governing spindle orientation^17^,^42^, at the concentration that suppressed phosphorylation of the substrate CrkL efficiently (Fig. 4n, P-CrkL), the cortical localization of caveolin-1 was abolished in mitotic cells with no detectable change in the expression level of caveolin-1 protein (Fig. 4m,n). Notably, the intercellular compartment of caveolin-1 vesicles was obviously detected in all cases, indicating that β1-integrin-dependent cell–ECM adhesion is dispensable for the internalization of caveolin-1 during mitosis. These results demonstrate that, in cells cultured on an ECM, β1-integrin signals are a prerequisite for the localization of caveolin-1 within the CEMM at the retracting cortical area during mitotic cell rounding and subsequently at the proximal end of the retraction fibres during metaphase.

### Caveolin-1 couples external cues with internal cues

Next, we further investigated how the β1-integrin-caveolin-1 pathway may influence spindle orientation. As expected, depletion of β1-integrin by RNAi randomized the spindle orientation on the line pattern (Fig. 4g). We found that Gαi1 still formed a crescent on the cell cortex proximal to the spindle poles in these cells (Fig. 4d,h). Furthermore, the cortical Gαi1 localization predicted spindle orientation by the cortical cue intensity-based prediction method in both control and β1-integrin-depleted cells (Fig. 4i). Similar results were obtained when the cells were treated with PP2 (Fig. 4j–l). Therefore, β1-integrin signalling is dispensable for Gαi1 crescent formation on the cell cortex proximal to the spindle poles, but is required for coupling the Gαi1 crescent with external cues of adhesion geometry.

Finally, we examined whether caveolin-1 plays a key role in the transmission of β1-integrin signalling to spindle orientation machineries. On an L-pattern, spindles were aligned along the hypotenuse of the ‘L' with high efficiency in control cells, as reported previously^17^, but randomly oriented in cells depleted of caveolin-1 by RNAi (Fig. 4o). Similar to cells depleted of β1-integrin, Gαi1 still formed a crescent on the cell cortex proximal to the spindle poles (Fig. 4p), and Gαi1 cortical localization predicted spindle orientation in both control and caveolin-1-depleted cells (Fig. 4o–q). Furthermore, expression of an siRNA-resistant form of GFP-caveolin-1 in caveolin-1-depleted cells restored the proper spindle orientation and correct positioning of the cortical Gαi1 crescent relative to the ECM geometry (Fig. 4o–r). Based on these results, we concluded that caveolin-1 provides the platform that links ECM-derived external cues and Gαi1–LGN–NuMA-dependent spindle orientation machineries to set the spindle orientation in the context of adhesion geometry.

It is noteworthy that Gαi1 still formed a crescent on the cell cortex even when the cells were treated with the Gαi1-activating reagent mastoparan-7 or Gαi1 inhibitor pertussis toxin (Supplementary Fig. 6; left, FN:Mas7 and FN:PT). This result indicated that the crescent formation is independent of the activity of Gαi1. A previous report has shown that cortical localization of NuMA and dynein is regulated by the position of the bipolar spindle^21^. Consistently, when the cells were treated with the Eg5 kinesin inhibitor S-trityl-l-cysteine (STLC) to induce monopolar spindles, Gαi1 no longer formed a crescent and was localized throughout the cortex. In addition, Gαi1 formed a crescent even when the cells were cultured on poly-L-lysine, but it was dispersed on the cortex when the cells are treated with STLC (Supplementary Fig. 6; right, PLL and PLL:STLC). Addition of Mas7 or pertussis toxin did not restore the Gαi1 crescent formation in STLC-treated cells (Supplementary Fig. 6; right, PLL:Mas7/STLC and PLL:PT/STLC). Therefore, a bipolar spindle, which regulates cortical localization of NuMA and dynein, is also necessary for the formation of the Gαi1 crescent on the cortex. It is interesting to speculate that a mitotic spindle generates the self-organised Gαi1-NuMA-dynein-mediated cortical cues, and then orientates toward the adhesion geometry through a Gαi1-caveolin-1 module.

## Discussion

**Figure 5:**
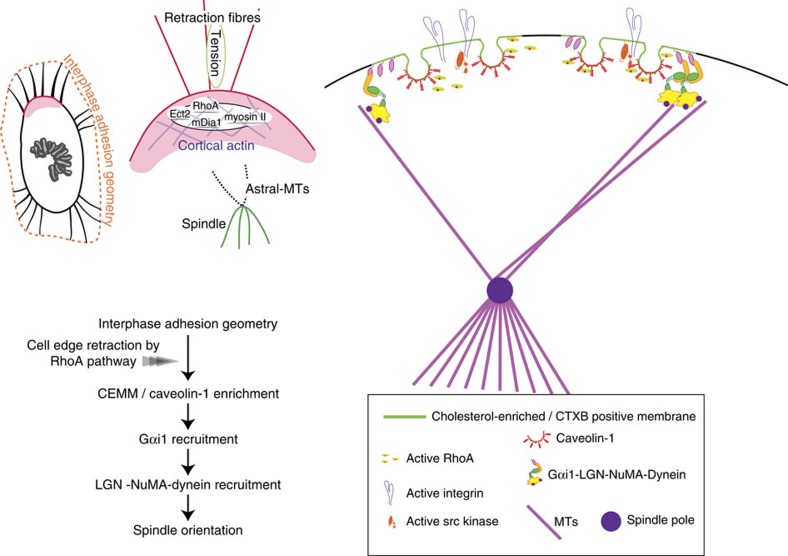
A model for the mechanisms that translate interphase adhesion geometry to the mitotic spindle orientation. The translation starts at the onset of mitotic cell rounding, when the heterogeneity of interphase adhesion geometry leads to anisotropic activation of RhoA. The regions with high RhoA activation rapidly retract the cell edge, and generate the caveolin-1-associated CEMM at the proximal end of retraction fibres. Gαi1 is preferentially localized within the CEMM that guides the Gαi1 cortical crescent to the proximal end of the retraction fibres. Consequently, Gαi1/LGN/NuMA complexes direct the spindle axis towards the cortex enriched with retraction fibres.

Although theoretical and physical studies implicate the spindle orientation in response to adhesion geometry, the molecular mechanisms that translate interphase geometry to the mitotic spindle orientation have remained unresolved. Figure 5 summarizes our working model for this translation. The translation starts at the onset of mitotic cell rounding, when the heterogeneity of interphase adhesion geometry, which may govern the distribution of focal adhesions, leads to anisotropic activation of RhoA. The regions with high RhoA activation rapidly retract the cell edge, probably through the ROCK-myosin II pathway and mDia, and gather the residual materials including disassembled focal adhesion molecules, which in turn generate the caveolin-1-associated CEMM enriched with GM1, active RhoA and active β1-integrin at the proximal end of retraction fibres. Gαi1 may be preferentially localized within the CEMM that guides the Gαi1 cortical crescent to the proximal end of the retraction fibres. Consequently, Gαi1/LGN/NuMA complexes may direct the spindle axis toward the cortex enriched with retraction fibres.

The mechanism that generates caveolin-1-associated CEMMs at the proximal end of retraction fibres in mitotic cells might relevant to the CEMM dynamics in migrating interphase cells. Previous studies have demonstrated that integrin signalling prevents internalization of CEMMs in migrating cells and targets GTP-bound (active)-RhoA/Rac1 to CEMMs to couple them to their effectors including mDia1 (refs 43, 44). Our results show that active β1-integrin localizes within the CEMM at the proximal end of retraction fibres, and that β1-integrin signals are required for the cortical localization of caveolin-1 in mitotic cells. These results support the model in which β1-integrin signals, probably including Src, prevent internalization of CEMMs, facilitating the cortical retention of caveolin-1 in mitotic cells. It is also noteworthy that phosphorylation of caveolin-1 on Tyr14 has been reported to mediate the internalization of CEMMs upon cell detachment^45^. Interestingly, phospho Tyr14-caveolin-1 signals were barely detected on the cortex in mitotic cells, even though the signals of both caveolin-1 and phospho Tyr14-caveolin-1 were obvious in interphase cells (Supplementary Fig. 7d). Thus, phosphorylation of caveolin-1 on Tyr14 is suppressed at the beginning of mitosis, and β1-integrin signals might be involved in this mechanism. It has been reported that ABL1 phosphorylates caveolin-1 on Tyr14 (ref. 46). In addition, our previous study has shown that ABL1 regulates spindle orientation in adherent cells^15^. Therefore, it is possible that ABL1-catalized phosphorylation of caveolin-1 on Y14 regulates the cortical localization or internalization of caveolin-1 during mitosis. However, siRNA-mediated knockdown of ABL1 led to no detectable defects in the cortical distribution or internalization of caveolin-1 in mitotic cells (Supplementary Fig. 7a,b). In addition, an unphosophorylatable mutant form of caveolin-1 (caveolin-Y14F) properly localized to the retracting cortical area during mitotic cell rounding (Supplementary Fig. 7c). Taken together, ABL1-mediated phosphorylation of caveolin-1 is unlikely to be involved in the cortical accumulation of caveolin-1 in mitotic cells. In our previous study, we show that ABL1-mediated phosphorylation of NuMA is required for its cortical anchoring during metaphase, but is dispensable for its cortical recruitment during prometaphase. We speculate that ABL1 is not involved in the cortical recruitment of spindle orientation regulators, including caveolin-1 and NuMA, during mitotic cell rounding in prometaphase, rather it is required for cortical anchoring of NuMA in metaphase.

A recent study by Kwon *et al.* has demonstrated that Myosin-10 accumulates in retraction fibres and asymmetrically localizes at the cortex to orient the spindles towards the retraction fibres through subcortical actin clouds^47^. Furthermore, our previous study has shown that Myosin-10 is concentrated at the tips and along the retraction fibres^16^, although we could not detect its asymmetric localization at the cortex, which may due to the differences in antibodies or fixation methods. We have previously reported that the binding of Myosin-10 to β1-integrin is required for β1-integrin-mediated spindle orientation control. Because active β1-integrin accumulates within the CEMM at the proximal end of retraction fibres (Fig. 4a,b), it is possible that the Myosin-10/β1-integrin complex might be recruited to the retracting cortical regions where it guides centrosomes towards subcortical actin clouds that are possibly induced by RhoA. From a comprehensive perspective, it makes sense that the RhoA/subcortical actin/Myosin-10-mediated microtubule-capturing mechanisms, together with caveolin-1/CEMM/Gα/LGN/NuMA-dependent microtubule pulling forces, set the spindle orientation in the context of adhesion geometry.

The molecules in these mechanisms have been shown to be necessary for the response to mechanical stress in directional cell movement^48^. Therefore, tension on a tissue might affect the spindle orientation through integrins, Rho and caveolin-1 interactions. During the revision of this manuscript, Bosveld and colleagues reported in the *Drosophila* epithelium that tricellular junctions function as landmarks of interphase cell shape anisotropy to orient spindles during mitotic cell rounding, which could account for orientation of division along mechanical strain axis^36^. Mitotic cell rounding in tissues being a fundamental mechanism during epithelial morphogenesis^49^,^50^, the mechanisms transmit interphase adhesion geometry or cell shape anisotropy to the spindle orientation during mitotic cell rounding might thus play a key role in tissue architecture modulated by mechanical constraints.

## Methods

### Cell culture, reagents and antibodies

HeLa cells, HeLa cells stably expressing GFP and RFP-H2B (HeLa-GFP-RFP-H2B), GFP-caveolin-1 (HeLa-GFP-caveolin-1), myristoylated GFP (HeLa-GFP-Myr), and RhoA Raichu FRET probe (HeLa-RhoA-Raichu) were cultured in Dulbecco's modified Eagle's medium with 10% fetal bovine serum. In all experiments, the cells were cultured on fibronectin and synchronized by a double thymidine block. Mitotically arrested cells were obtained by treatment with 10 μM STLC for 16 h after single thymidine block release.

The antibodies used included anti-Gαi1 (for staining, mouse, Santa Cruz Biotechnology, sc-56536, × 100; for western blotting, rabbit, Novus, NBP-1–31601, × 1,000), anti-caveolin-1 (rabbit, BD Pharmingen, #610059, × 200), anti-p-CrkL (rabbit, Cell Signaling Technology, #3181, × 1,000), anti-CrkL (rabbit, Santa Cruz Biotechnology, sc-319, × 500), anti-GFP (mouse, Clontech, #632569, × 2,000), anti-active-β1-integrin (mouse, AbD serotec, MCA2028, × 200), anti-β1-integrin (rabbit, Cell Signaling Technology, #9699, × 200), anti-α-tubulin (mouse, Sigma, DM1A, × 10,000), anti-mDia1 (mouse, BD Pharmingen, #610848, × 200), anti-Ect2 (rabbit, BETHYL, A302–348A, × 200), anti-RhoA (mouse, Santa Cruz Biotechnology, sc-418, × 200), anti-HA (mouse, Roche, 12CA5, × 200), anti-ABL1 (mouse, Calbiochem, OP20, × 50). For live imaging and staining, living cells were incubated with anti-active-β1-integrin antibodies labelled with a Zenon Alexa Fluor 546 Mouse IgG Labeling kit (Molecular Probes, Z25004) or FITC conjugated Cholera Toxin B Subunit (Sigma, #C1655, × 100).

### siRNA experiments

Oligonucleotides were purchased from Japan Bioservice. The siRNA sequences were as follows: human caveolin-1, 5′-GAGCUUCCUGAUUGAGAUUtt-3′; human RhoA-1si (ref. 51), 5′-GAAGUCAAGCAUUUCUGUCtt-3′; human RhoA-2si (ref. 52), 5′-CAGAUACCGAUGUUAUACUtt-3′; human Ect2-1si (ref. 23), 5′-UUGCCUAGAGAUAGCAAGAtt-3′; human Ect2-2si (ref. 23), 5′-AUGACGCAUAUUAAUGAGGAUtt-3′; human mDia1-1si (ref. 53), 5′-GCUGGUCAGAGCCAUGGAUtt-3′; human mDia1-2si (ref. 54), 5′-CAGCCGCUGCUGGAUGGAUtt-3′; human β1-integrin, 5′-GGAAUGCCUACUUCUGCACtt-3′. HeLa cells were transfected with siRNA for 4 h using Oligofectamine (Invitrogen) and then synchronized by a double thymidine block. The expression levels of caveolin-1, RhoA, Ect2, mDia1 and β1-integrin were analysed by western blotting. Full size images of western blotting were shown in Supplementary Figs 8 and 9.

### Plasmid constructs and transfection

Full-length human caveolin-1 cDNA was cloned into pEGFP N1 and pDsRed monomer N1. pMyrPalm_mEGFP_IRES_puro2b was a gift from Daniel Gerlich (Addgene plasmid # 21038)^55^. siRNA-resistant GFP-caveolin-1 was constructed by site-directed mutagenesis. HeLa cells were transfected for 1 h with plasmids using Lipofectamine Plus Reagent (Invitrogen) after the first release from the double thymidine block and then washed with fresh medium.

### Immunoprecipitation

Cells were lysed in buffer A (50 mM Hepes (pH 7.4), 10 mM 2-glycerophosphate, 50 mM NaCl, 2 mM MgCl_2_, 2 mM EGTA, 1 mM EDTA, 15 mM NaF, 10% glycerol, 0.5% NP-40, 5 mM dithiothreitol, 1 mM phenylmethylsulfonyl fluoride, 5 mM Na_3_VO_4_ and 2 μg ml^−1^ aprotinin) and centrifuged at 20,000*g* for 15 min. The exogenously expressed GFP or GFP-caveolin-1 was immunoprecipitated with anti-GFP antibodies-conjugated beads (Nacalai; 06083-05). After 3 h incubation at 4 h, beads were washed three times with buffer B (buffer A without NP-40 and NaCl).

### Time-lapse imaging

Cells were plated onto a 35-mm glass-based dish (IWAKI) or 2-well chambered coverglass (IWAKI) in medium with 20 mM HEPES (pH 7.3). For imaging analysis, HeLa cells expressing GFP and RFP-H2B were cultured on a fibronectin-coated dish, and time-lapse images were taken every 2 min. Images were obtained by an Olympus IX81-ZDC microscope and 40 × objective with a temperature-controlled and motorized stage and Meta Morph software (Molecular Devices).

### Cell staining

For detection of Gαi1 and caveolin-1, cells were fixed with 4% paraformaldehyde followed by incubation with methanol at −20 °C for 10 min or 0.2% Triton X-100 at room temperature for 10 min. For detection of active β1-integrin, cells were incubated with medium including an anti-active β1-integrin antibody at 4 °C for 5 min. Subsequently, the cells were fixed with 4% paraformaldehyde followed by incubation with 0.2% Triton X-100 at room temperature for 10 min. The cells were blocked with 5% bovine serum albumin at 37 °C for 30 min, incubated with primary antibodies at 4 °C overnight, washed, and then incubated for 1 h with secondary antibodies (Alexa Fluor 488- or 594 goat anti-mouse, anti-rabbit or anti-rat IgG (Molecular Probes)). Fluorescent labelling was visualized using an inverted wide-field fluorescence microscope (IX81-ZDC, Olympus, Japan). Three-dimensional images (0.2 μm intervals) were obtained using an Olympus oil immersion objective lens (UPLSAPO 60XO/NA1.35) and computationally processed by three-dimensional deconvolution using Meta Morph software. For Figs 1i, 3b,d and 4a,b, images were obtained using an inverted confocal fluorescence microscope (Leica Microsystems, TCS SP8 with Hybrid Detector) and LAS-X software. Images were analysed using Image J software.

### Spindle orientation analysis

Images of mitotic cells were obtained using the inverted wide-field fluorescence microscope and analysed for the spindle angle against a respective micropattern using Image J software. Spindle angles were calculated as the orthogonal oriented angle of aligned chromosomes. To quantify the cortical intensity of caveolin-1 or Gαi1, we first extracted the cellular edge region using a manual thresholding, and then the signal intensity for each angle from the centre within the edge region was was measured by using ImageJ ‘line' command. We normalized the intensity by dividing the raw intensity with the sum of the intensity of all angles. The cortical intensity-based prediction was conducted as explained in the ‘Theoretical analysis of spindle orientation' except the forces pulling a pole of the spindle at a given direction are proportional to the signal intensity of caveolin-1 or Gαi1.

### Correlative light and electron microscopy

Correlative light and electron microscopy imaging was performed according to a method described previously^35^ with some modification. HeLa cells stably expressing GFP-caveolin-1 were cultured in a special glass bottom dish with an addressing grid (grid size: 50 μm) on the coverslip (ibidi, Munich, Germany). Cells were fixed with 2.5% glutaraldehyde for 1 h. After staining with Hoechst 33342, computationally processed three-dimensional images (60 focal planes at 0.2 μm intervals) were obtained using an IX81-ZDC microscope. Electron microscopy observation of the same cell was carried out as follows. Cells were post-fixed with 1% OsO_4_ (Nisshin EM Inc., Tokyo, Japan) in phosphate buffer (pH 7.4) for 1 h, washed briefly with distilled water, and then stained with 2% uranyl acetate (Wako, Tokyo, Japan) for 1 h. Then, the samples were sequentially dehydrated with 30, 50, 70, 90 and 100% ethanol, and embedded in epoxy resin by incubating sequentially with 10% and 30% and 50% and 70% (V/V) Epon812 (TAAB, Berkshire, UK) in ethanol for 3 min, 90% Epon812 for 10 min, and 100% Epon812 for 1 h. The epoxy block containing the same cell observed under a fluorescence microscope was trimmed according to the address on the coverslip and cut into ultrathin sections (80 nm) by a microtome (Leica Microsystems, Wetzlar, Germany). The sections were stained with 4% uranyl acetate (Wako, Tokyo, Japan) for 20 min and then a commercial ready-to-use solution of lead citrate (Sigma-Aldrich, St Louis, USA) for 1 min. Image data were collected by an electron microscope (JEM1400, 120 kV, JEOL, Tokyo, Japan).

### Theoretical analysis of spindle orientation

In our image processing algorithm, the shapes of the cell and nucleus were determined through binarisation of GFP and RFP-H2B images, respectively^56^. For the binarization, Otsu's method was used after applying Navier–Stokes based image inpainting method^57^ to remove non-target cell regions. The centre of the nucleus was determined as the centroid of the nucleus, and the distance, Dis(*θ*), between the nucleus centre and the edge of the cell was measured every 1°. The timing of cell rounding was automatically determined as the time point with the largest decrease in the mean value of Dis(*θ*) from the previous time point, which was confirmed and revised manually if necessary. The aspect ratio of the cell was calculated as that of an oval with the best fit to the cell shape. After nuclear envelope breakdown, the RFP-H2B signal indicated mitotic chromosomes. At metaphase, the linear fitting of the binarized chromosome signals provides the angle of the metaphase plate. At anaphase, the fitting provides the angle of chromosome segregation. Because these two angles are almost perpendicular, we could automatically detect the timing of anaphase onset as the time point at which the inner product of the chromosome angle with the angle of the previous time point is the minimum. The chromosome angle at anaphase onset is defined as the angle of the cell division axis at anaphase onset. We have noticed that the chromosome angle sometimes changes dynamically after anaphase onset, and we defined the chromosome angle at three frames (that is, 6 min) after anaphase onset as the cell division axis at anaphase rescue. The angles at anaphase onset and rescue differed as much as 30°.

We predicted the cell division axis from the cell shape by calculating the potential energy landscape as developed by Théry *et al.*^19^. In the original definition, the forces pulling a pole of the spindle at a given direction are proportional to the contour length of the cell at the direction, and thus proportional to the distance between the pole and the edge of the cell at the direction. In this study, for the simplicity of the calculation, we approximated the pole-to-edge distance to the distance between the centre of the nucleus and the edge of the cell at the direction. This approximation does not affect the magnitude relationship of the energy among the angles, and thus does not affect the value of the predicted angle that gives the minimum energy. We assumed each of the two spindle poles receives forces from each half of the cell area divided by the line perpendicular to the spindle, which passes through the midpoint of the spindle. By summing the pulling forces acting on each pole from every 1°, the torque acting on the spindle and the potential energy landscape was calculated as described previously^19^. The unit of the energy is an arbitrary unit. The predicted direction of the cell division axis is the angle that minimizes the potential energy landscape. We also calculated the energy landscape based on retraction activity, assuming the pulling forces proportional to the velocity of decrease in the distance between the nuclear centre and the cellular edge.

### Polyacrylamide hydrogel preparation

Polyacrylamide hydrogels were prepared on 35-mm glass-based dishes (IWAKI) as previously described^58^. Briefly, a glass-based dish was treated with 0.1 N NaOH and then 3-aminopropyltrithoxysilane, rinsed with dH_2_O, and incubated in 0.5% glutaraldehyde/PBS for 30 min. Subsequently, acrylamide (12%)/bis-acrylamide (0.25%) mixtures were polymerized between the glass-based dish and a coverslip, washed with dH_2_O, incubated with 0.25 mg ml^−1^ Sulpho-SANPAH (Thermo Scientific Pierce) in HEPES buffer under 365 nm ultraviolet light for 10 min, and then washed with dH_2_O. Then, the gel was incubated with a 10 μg ml^−1^ fibronectin solution (Sigma-Aldrich) in HEPES buffer, followed by two rinses with medium.

### Statistical analysis

Quantitative data were analysed statistically (mean and s.d.). Angular distributions were compared using the non-parametric test of Kolmogorov–Smirnov in the R statistical environment.

### Data availability

The data that support the findings of this study are available from the corresponding author upon request.

## Additional information

**How to cite this article:** Matsumura, S. *et al.* Interphase adhesion geometry is transmitted to an internal regulator for spindle orientation via caveolin-1. *Nat. Commun.* 7:11858 doi: 10.1038/ncomms11858 (2016).

## Supplementary Material

Supplementary Figures Supplementary Figures 1-9

Supplementary Movie 1 Time-lapse images of mitotic HeLa cells expressing GFP-caveolin-1 and RFP-histone H1. Time interval is 2 min.

Supplementary Movie 2 Time-lapse images of mitotic HeLa cells expressing Myr-GFP and RFP-caveolin-1 on a fibronctin-coated glass bottom dish. Time interval is 2 min.

Supplementary Movie 3 Time-lapse images of mitotic HeLa cells expressing Myr-GFP and RFP-caveolin-1 on fibronctin-coated 12 % polyacrylamide gels. Time interval is 2 min.

Supplementary Movie 4 Time-lapse images of mitotic HeLa cells expressing GFP-caveolin-1 on a fibronctin-coated glass bottom dish. To visualize activeβ1 intgrin, Alexa 546-labelled anti-active β1 intgrin antibody was used. Time interval is 2 min.
