# Supplementary material for: Interphase adhesion geometry is transmitted to an internal regulator for spindle orientation via caveolin-1
Source: Nat Commun. 2016 Jun 13;7:ncomms11858. doi: 10.1038/ncomms11858 (PMC4910015; doi:10.1038/ncomms11858)
Supplement: Supplementary Figures — 1-9 [file ncomms11858-s1.pdf]

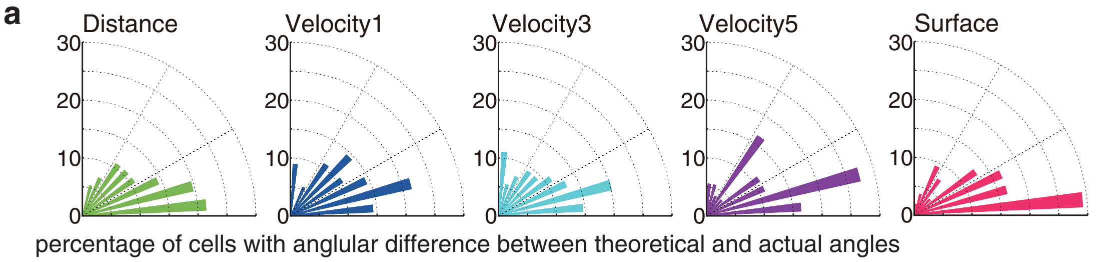

	Distance	Velocity 1	Velocity 3	Velocity 5	Surface
Size (n)	55	55	55	55	55
Mean Direction	31.0	32.6	34.9	37.7	28.1
Circular Variance	0.07	0.08	0.08	0.10	0.08
Circular S.D.	0.39	0.41	0.41	0.46	0.41

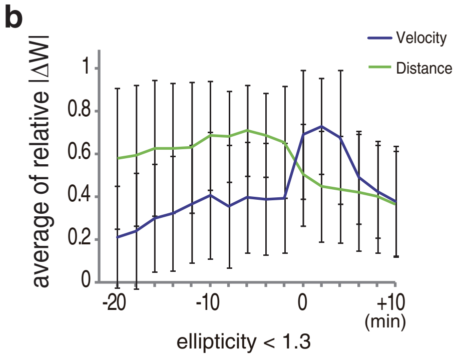

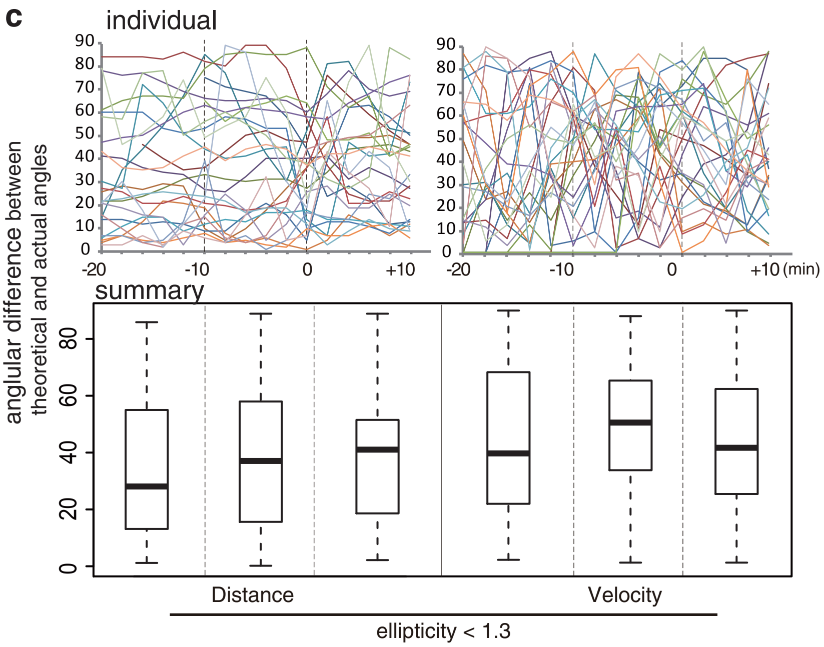

Supplementary Figure 1 The predicted angles by interphase adhesion geometory and by cell edge retraction during mitotic cell rounding.

(a) The theoretical angles from alternative methods show similar results. (Upper) Angular distribution (n = 55) of accuracy of the predicted spindle orientation. Each velocity was defined by interval of 2 min (1 inter-frame), 6 min (3 inter-frame) or 10 min (5 inter-frame) (Velocity 1, Velocity 3 and Velocity 5, respectively). (Bottom table) Values of sample size, Mean of the predicted angles, two sorts of variance value in the predicted angles. (b) The fold change of the average of ΔW at every 10 min (5 frames) in cells with less than 1.3 ellipticity. Time 0 represents the onset of cell rounding. (c) Angular difference between the theoretical and actual spindle angles in each time point in every sample (upper). The theoretical angles were calculated by Distance- (left) or Velocity-based method (right) in the cells with less than 1.3 ellipticity. Box plot representing the angles for every 10 min (5 frames) are shown in bottom.

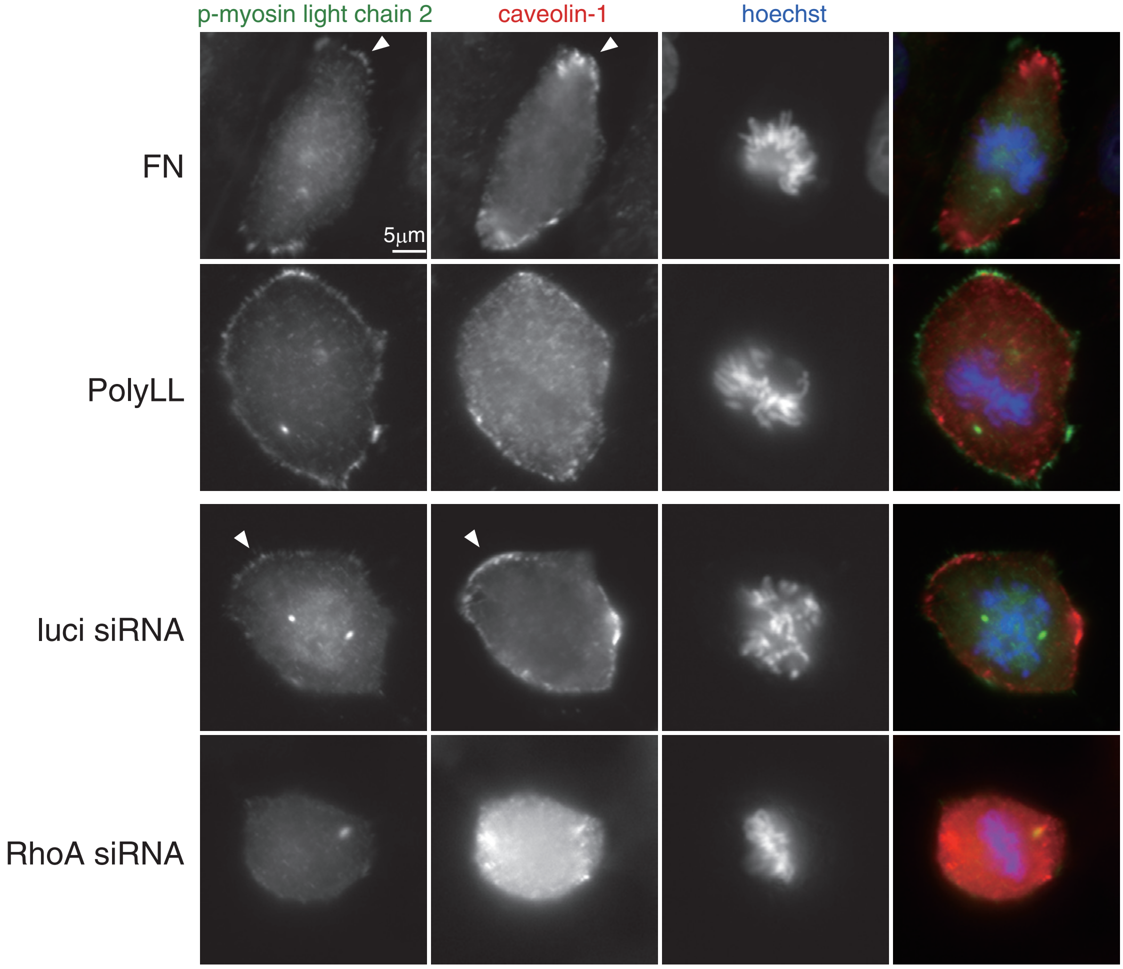

Supplementary Figure 2 Phosphorylation of myosin light chain2 localised at retracting cellular edge together with caveolin-1.

Images of prometaphase or metaphase cells, cultured on FN- or PolyLL-coated coverglass, or treated with RhoA siRNAs or control luci siRNA, and stained with anti-phospho myosin light chain 2 antibody (green), anti-caveolin-1 antibody (red) and Hoechst (blue). Phospho-myosin light chain 2 and caveolin-1 was enriched to the specific retracted cortical area in mitotsis (arrowhead).

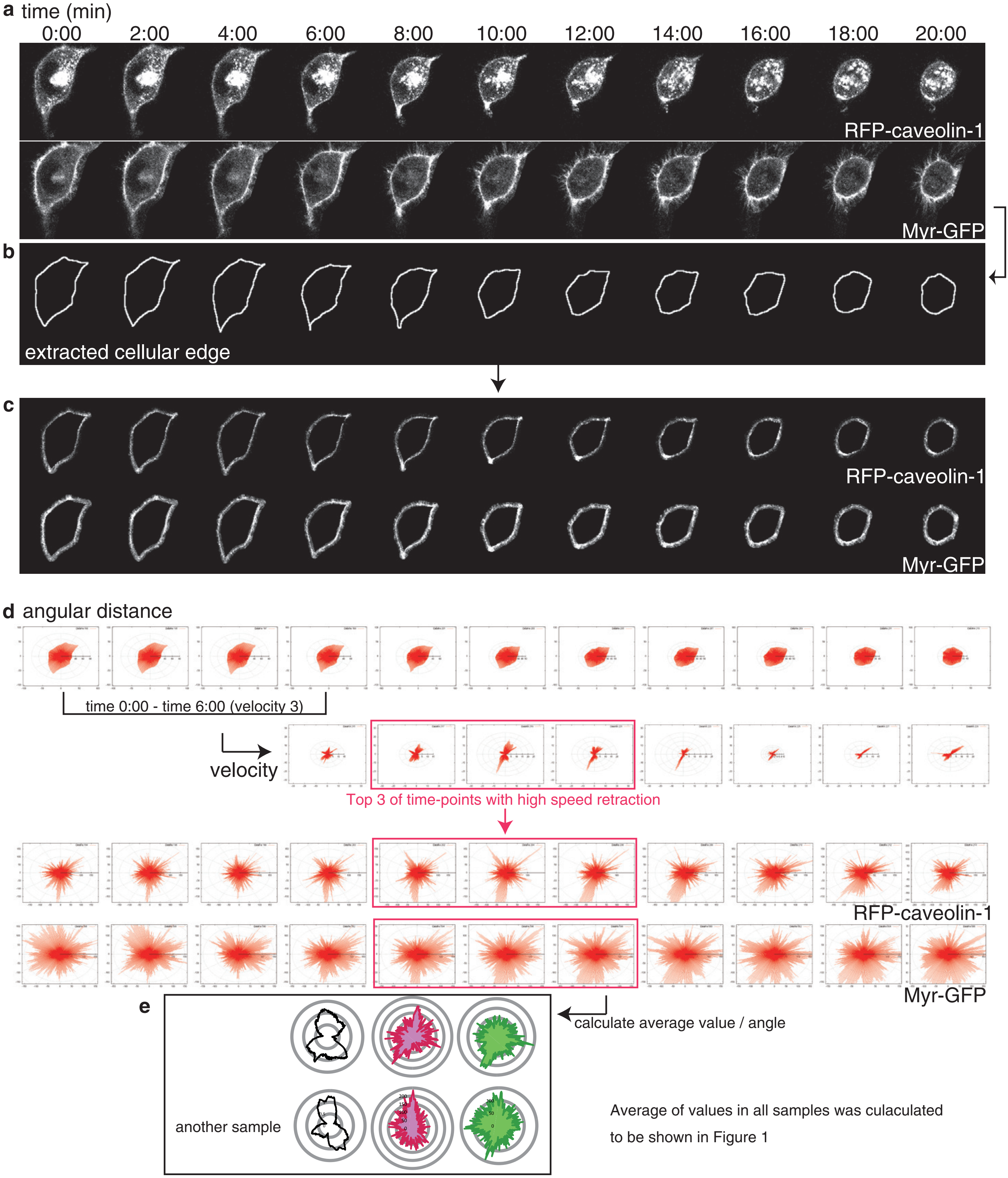

Supplementary Figure 3 Scheme of image analysis for cellular edge retraction and cortical signal intensity. (a) Timelapse images of a sample. (b) Extracted cellular edge. (c) Cropped images of cellular edge using (b). (d) Images of values per angle in polar coords. From data of velocity, "Top3 timepoints" were selected, which include a large number of the high value of velocity (using more than 70 % threshold). (e) Average value per angle from values in the selected timepoints was calculated. Finally, we calculated average values among all samples per angle.

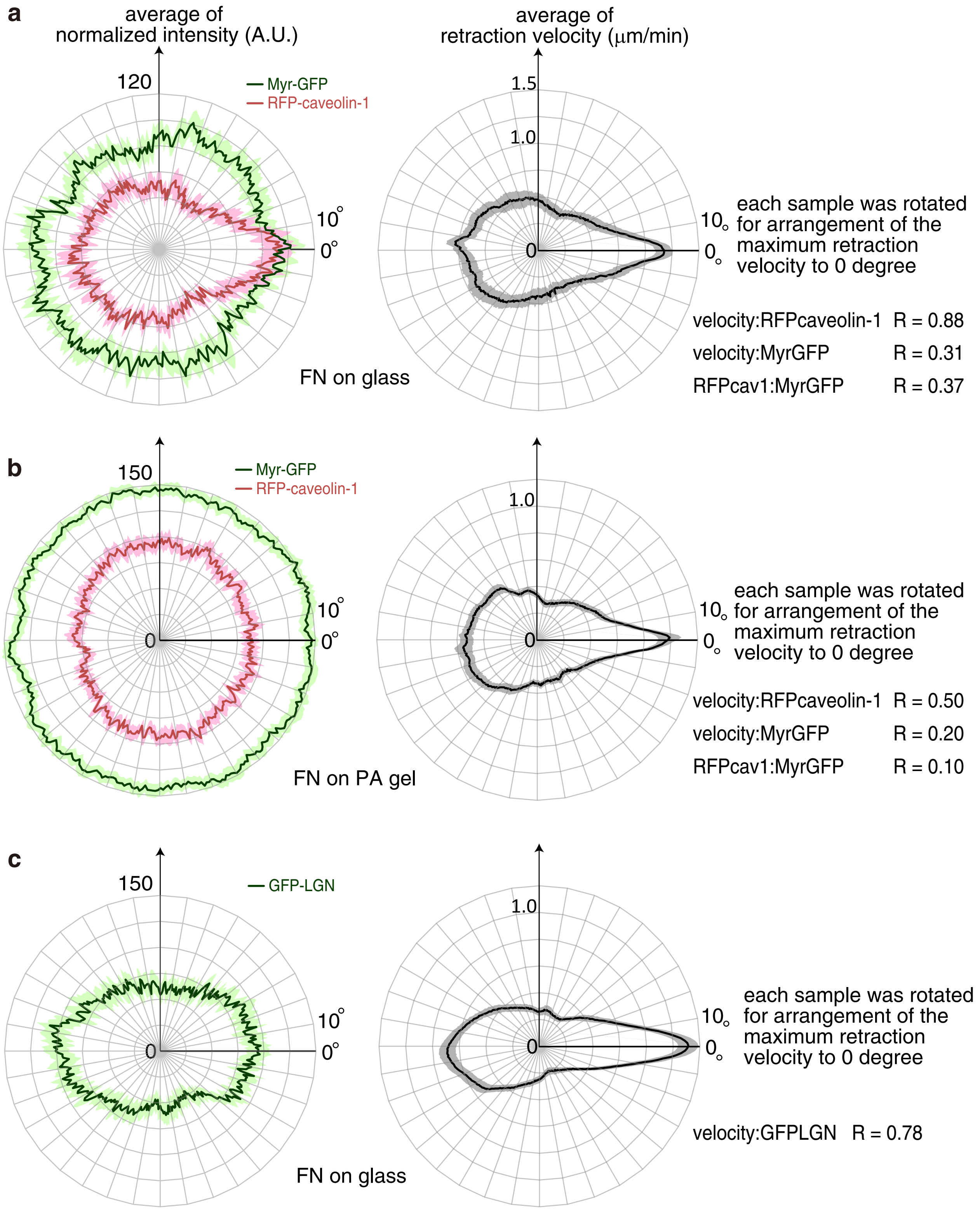

Supplementary Figure 4 Quantification analyses for the retraction velocity and cortical signal intensity. (a - c) Indicated distribution of average value per angle (average ± S.E.) (left) or distribution of average retraction velocity per angle (μm/min) (average ± S.E.). Maximum retraction velocity was arranged to 0 degree by rotation. n = 18 in (a), n = 38 in (b) and n = 38 (c). R indicates the Pearson correlation coefficient respectively.

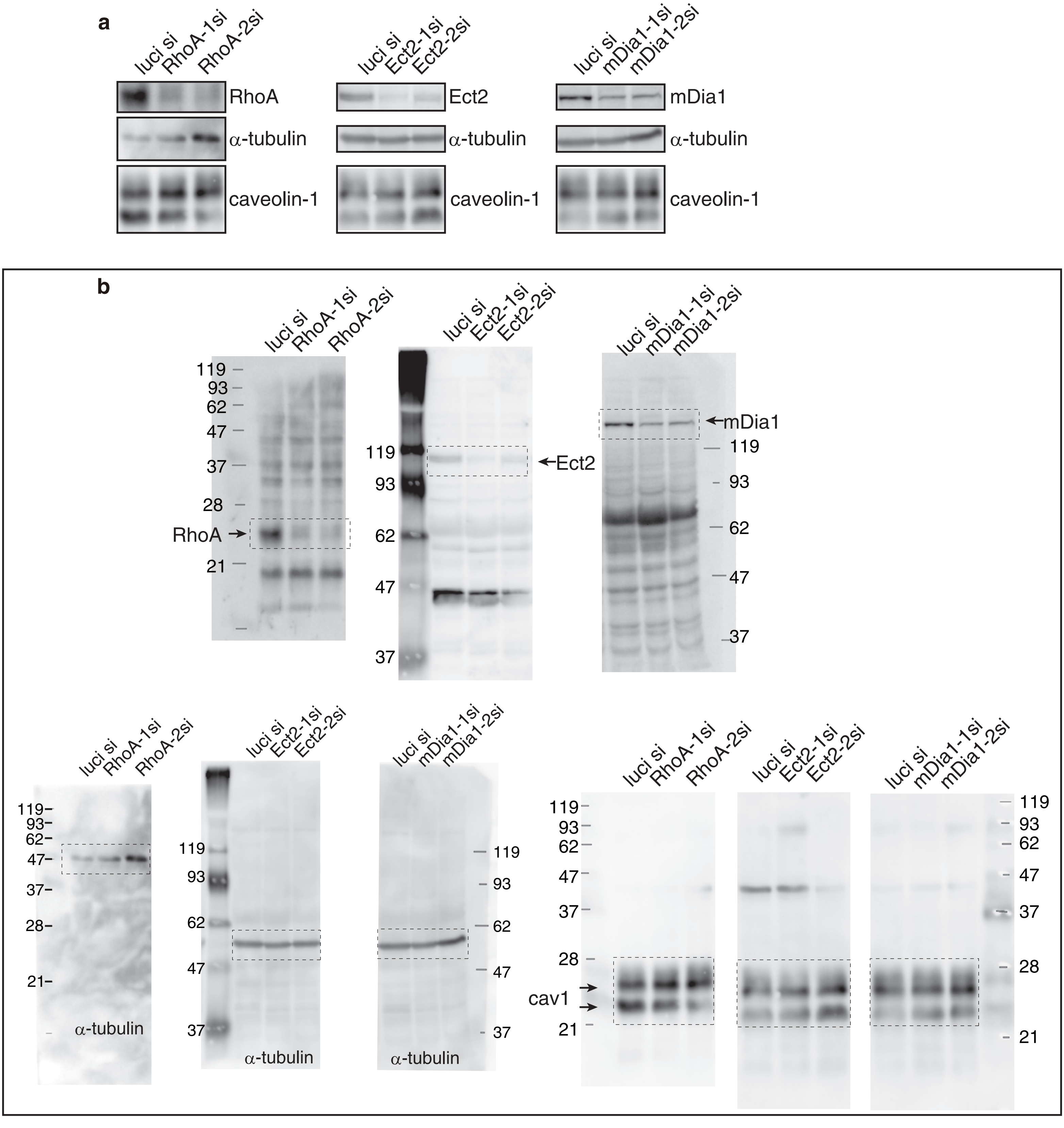

Supplementary Figure 5 Western blot analysis for siRNA treated cells.
(a) Western blot analysis of mDia1, Ect2, RhoA, caveolin-1 and control α-tubulin in the cells in Fig. 3b.
(b) Uncropped images of westernblotting in this figure.

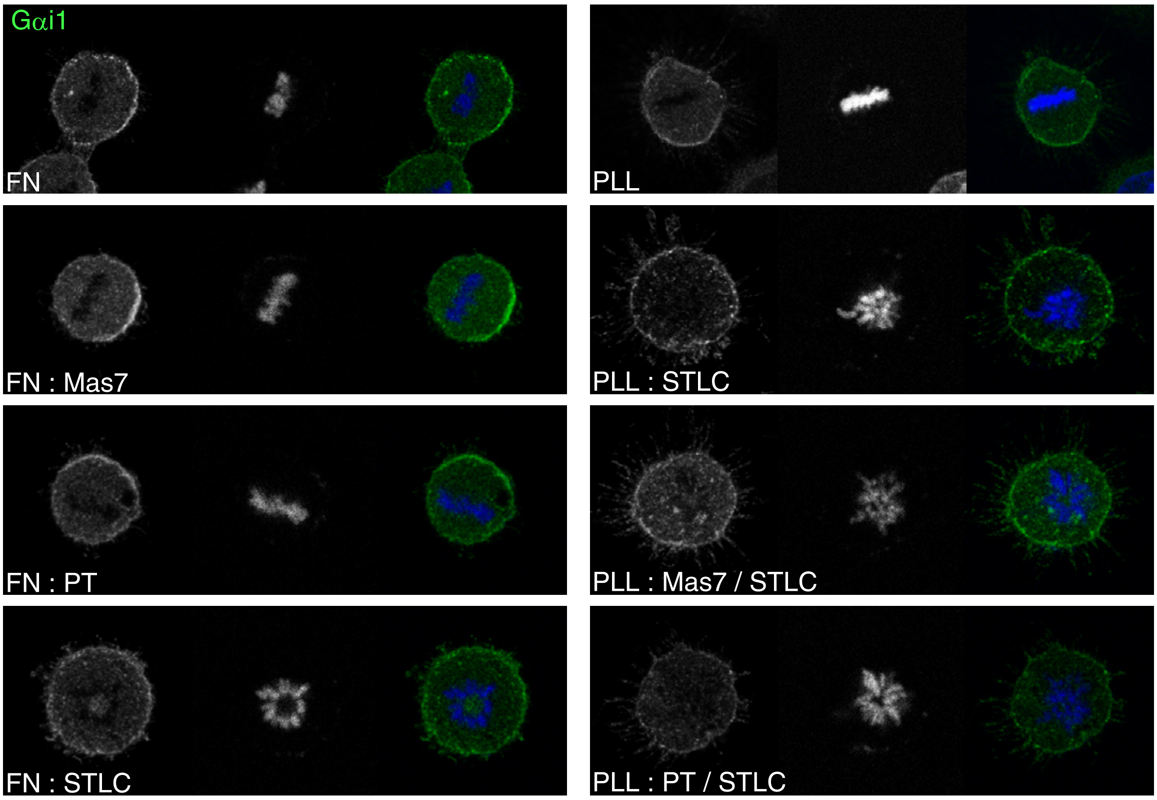

Supplementary Figure 6 Bipolar spindle formation and chromosome alignment, but not ECM-cell adhesion derived signals, are involved in the cortical crescent formation of Gαi1 in mitosis.

Images of mitotic cells stained with anti Gαi1 antibody and hoechst. Cells were cultured on indicated coverslip and cells were treated for 1 hr with indicated drugs (Mas7 (12.5 μM), PT (500 ng/ml) and STLC (10 μM)). FN, fibronectin; PLL, poly-L-lysine; Mas7, Mastoparan-7; PT, Pertussis toxin; STLC, S-trityl-L-cysteine.

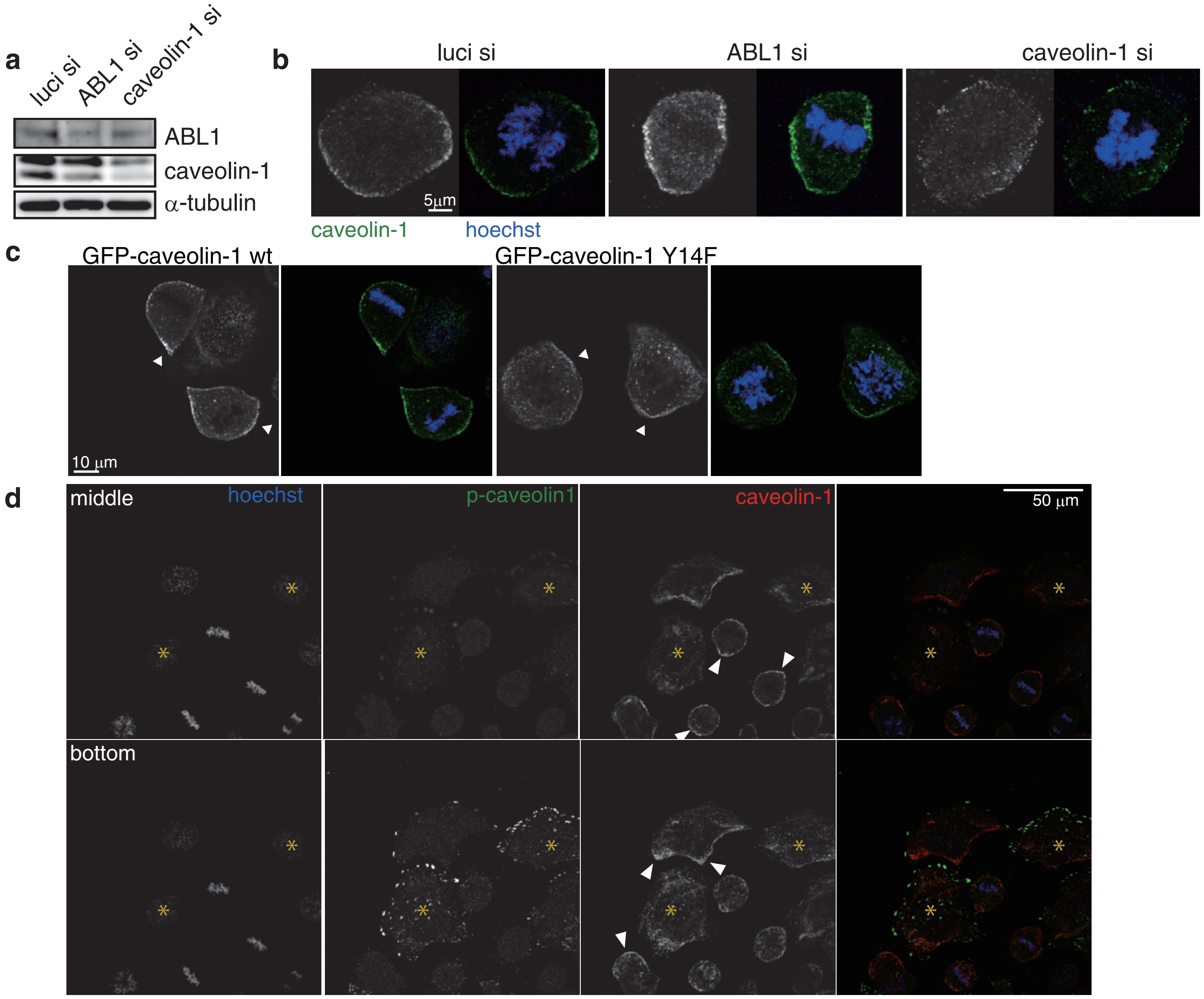

Supplementary Figure 7 Phosphorylation of caveolin-1 Y14 is not required for caveolin-1 cortical localization. (a) Western blot analysis of ABL1, caveolin-1 and control α-tubulin in the cells in (b). (b) Images of prometaphase cells, treated with ABL1 siRNAs, caveolin-1 siRNAs or control luci siRNA, and stained with anti-caveolin-1 antibody (green) and Hoechst (blue). (c) HeLa cells expressing either GFP-caveolin-1 wt or GFP-caveolin-1-Y14F mutant were cultured on fibronectin-coated coverslip. Cells were fixed and stained with hoechst 33342. Both GFP-caveolin-1 wt and Y14F were recruited to the specific cortical area in prometaphase/metaphse cells (arrowhead). (d) HeLa cells cultured on fibronectin-coated coverslip were synchronized in M phase. Cells were fixed and stained with anti-phospho-caveolin-1 antibody (mouse, BD), anti-caveolin-1 antibody and hoechst 33342. Phospho-caveolin-1 foci were disappeared and caveolin-1 was enriched to the specific retracted cortical area in mitotsis (arrowhead). Cells marked with yellow * were interphase cells.

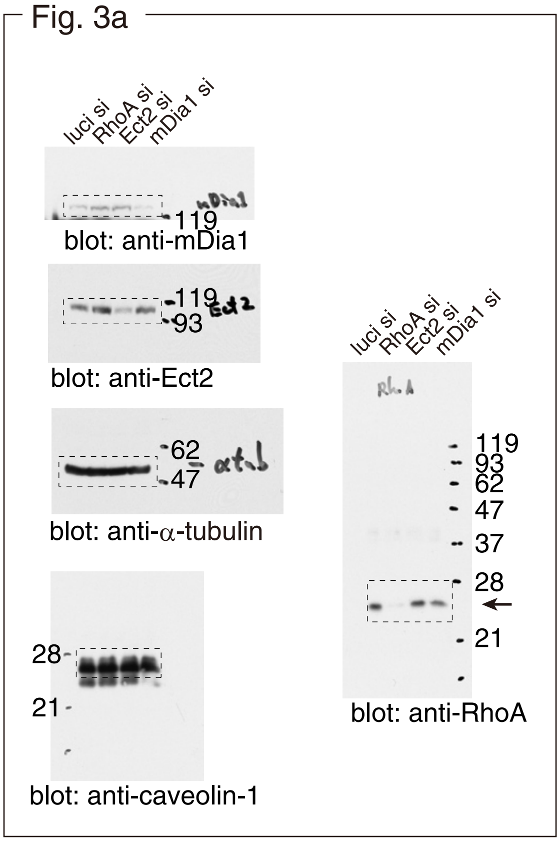

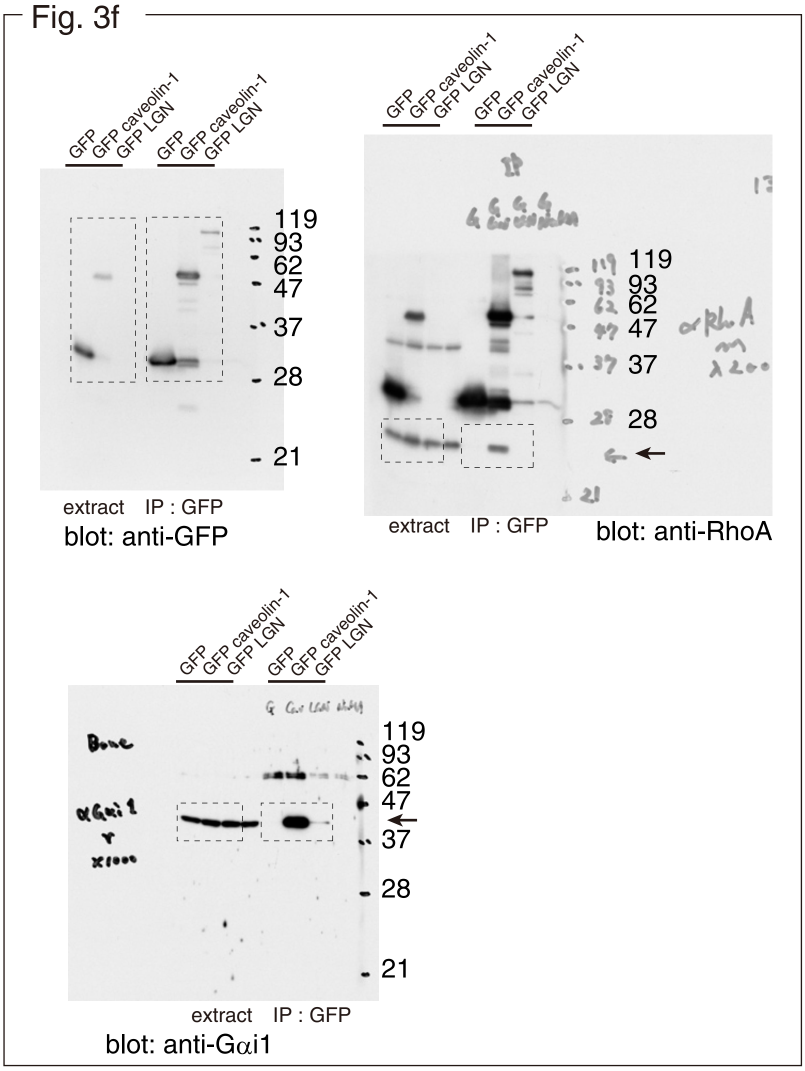

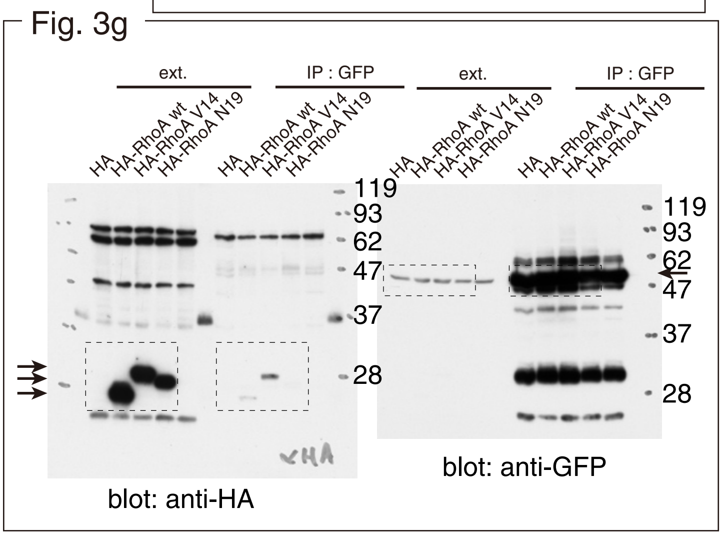

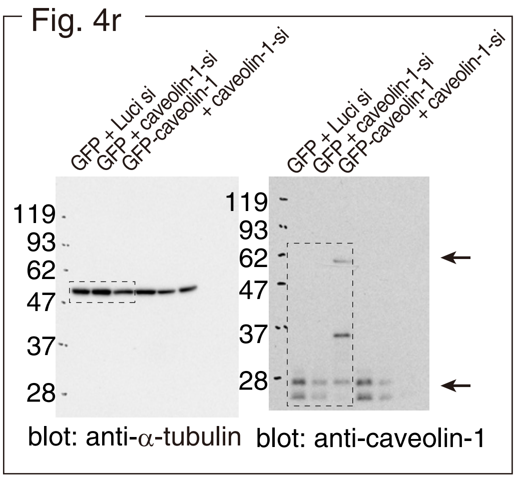

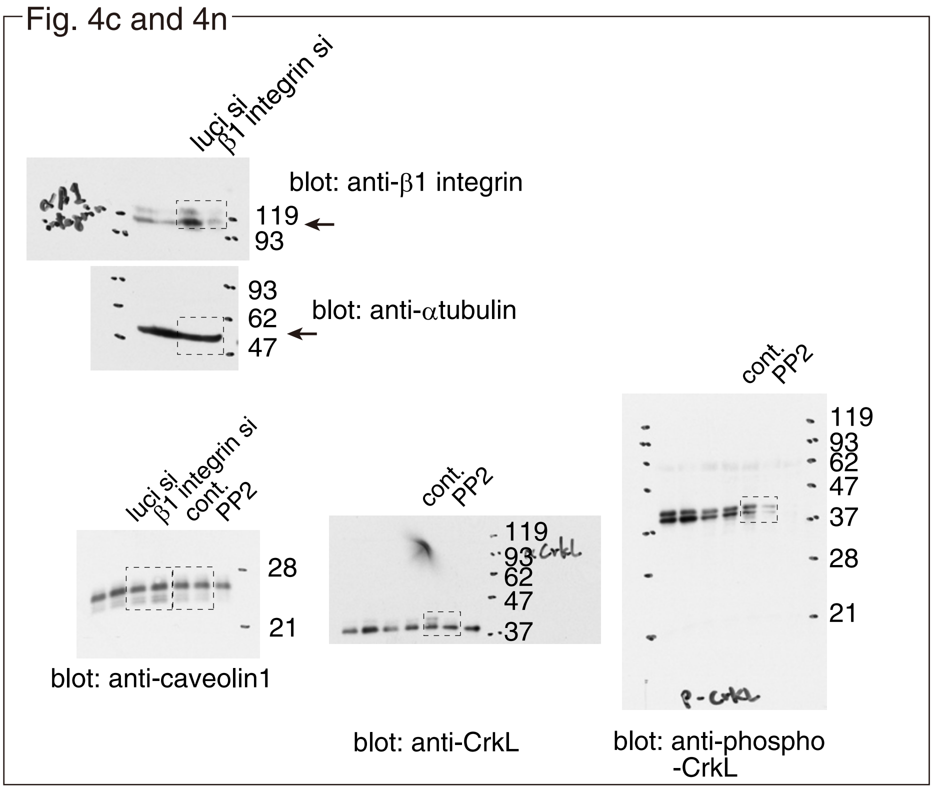

Supplementary Figure 8 Uncropped images of westernblotting.

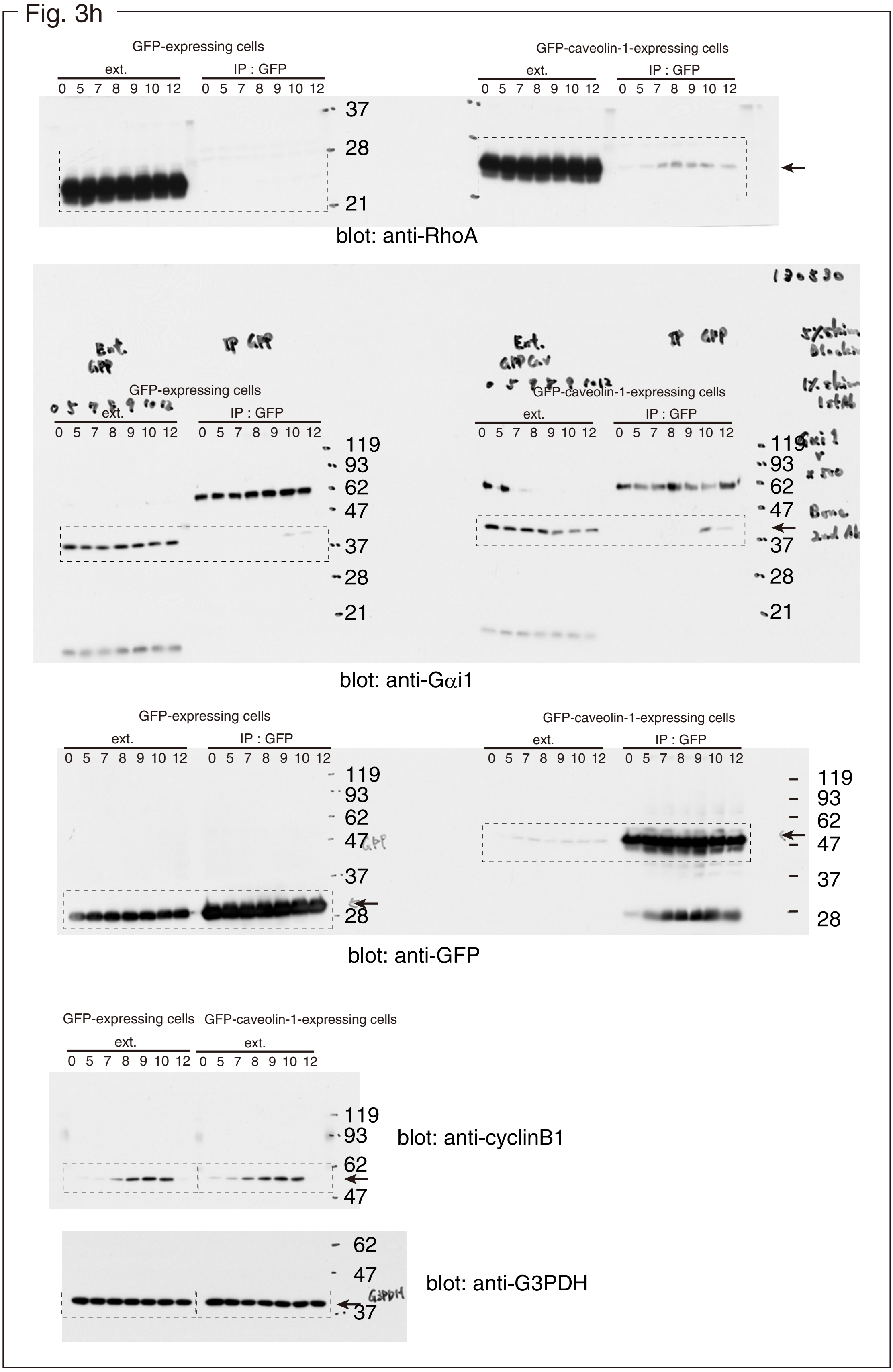

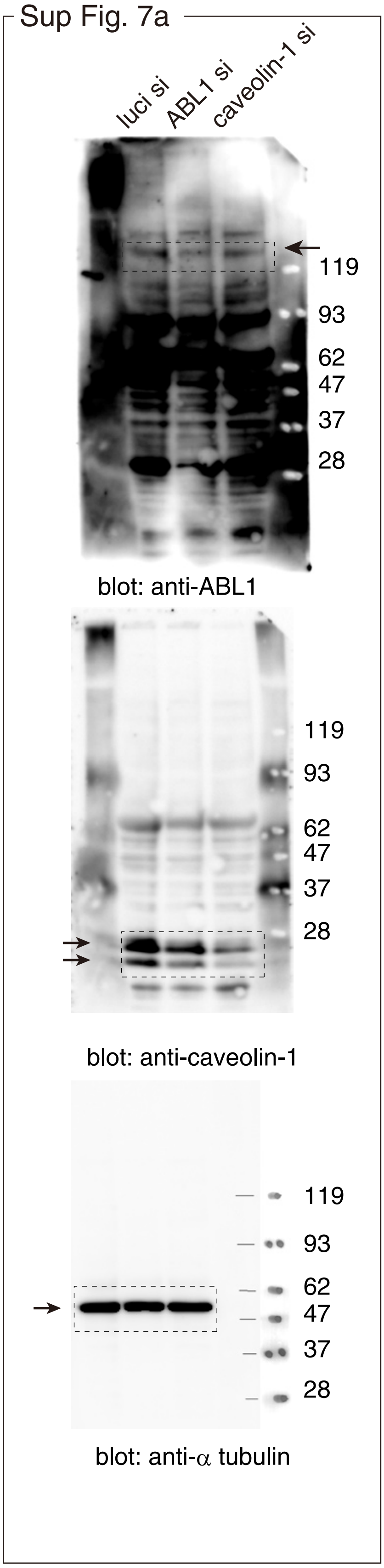

Supplementary Figure 9 Uncropped images of westernblotting.
